# Detection of H1 Swine Influenza A Virus Antibodies in Human Serum Samples by Age Group^1^

**DOI:** 10.3201/eid2609.191796

**Published:** 2020-09

**Authors:** Elien Vandoorn, Isabel Leroux-Roels, Geert Leroux-Roels, Anna Parys, Amy Vincent, Kristien Van Reeth

**Affiliations:** Ghent University, Merelbeke, Belgium (E. Vandoorn, A. Parys, K. Van Reeth);; Ghent University and Ghent University Hospital, Ghent, Belgium (I. Leroux-Roels, G. Leroux-Roels);; National Animal Disease Center, Ames, Iowa, USA (A. Vincent)

**Keywords:** influenza, humans, swine, hemagglutinin, antibodies, hemagglutination inhibition, pandemic, public health, viruses, zoonoses

## Abstract

Most H1 influenza A viruses (IAVs) of swine are derived from past human viruses. As human population immunity against these IAVs gradually decreases, the risk of reintroduction to humans increases. We examined 549 serum samples from persons 0–97 years of age collected in Belgium during 2017–2018 for hemagglutination inhibiting and virus neutralizing antibodies against 7 major H1 swine IAV (swIAV) clades and 3 human progenitor IAVs. Seroprevalence (titers >40) rates were >50% for classical swine and European human-like swIAVs, >24% for North American human-like δ1a and Asian avian-like swIAVs, and <10% for North American human-like δ1b and European avian-like swIAVs, but rates were age-dependent. Antibody titers against human-like swIAVs and supposed human precursor IAVs correlated with correlation coefficients of 0.30–0.86. Our serologic findings suggest that European avian-like, clade 1C.2.1, and North American human-like δ1b, clade 1B.2.2.2, H1 swIAVs pose the highest pandemic risk.

Humans and swine are susceptible to influenza A viruses (IAVs) of hemagglutinin (HA) subtypes H1 and H3, which are widespread in both species. Human IAVs frequently are transmitted to swine, after which the HA surface protein generally undergoes slower antigenic evolution (drift) in swine than in humans (*1*–*3*). Therefore, swine can be considered a reservoir for past human IAVs. Because antigenic drift variants of human IAVs replace each other over time, younger persons only have been exposed to more recent strains and human population immunity against older human IAVs gradually decreases (*4*). Consequently, human-origin swine IAVs (swIAVs) can be reintroduced into the human population after a certain period and cause a pandemic, as illustrated by the influenza A(H1N1)pdm09 virus (pH1N1) (*5*). The H1 of this swine-origin virus is related to the H1 of human seasonal H1N1 IAVs that circulated in 1918–1950. In 2009, only persons born before the 1950s had cross-reactive antibodies against pH1N1, so a pandemic was possible (*6*,*7*).

The evolution of swIAVs is different from and more complex than that of human IAVs because of multiple introductions of human IAVs into swine and geographic separation of swine populations (*8*). H1 swIAV colloquial names indicate their origin and region of circulation. An improved classification system subdivides H1 swIAVs into 3 lineages and 28 clades on the basis of H1 nucleotide sequence homology (*9*). The lineages are 1A, 1B, and 1C, with the number representing the subtype (H1) and the letter representing the lineage. Clades and subclades are indicated with 1–3 digits. Classical swine lineage 1A contains IAVs with the human 1918 pandemic H1N1 virus as a common ancestor. Most clades are restricted to America and Asia, but pH1N1 viruses (1A.3.3.2) circulate in swine and humans worldwide. Human seasonal lineage 1B contains swIAVs with an H1 derived from human seasonal IAVs. These human-like H1 swIAVs emerged in Europe in the late 1980s and in North America in the early 2000s. Eurasian avian lineage 1C contains swIAVs that originated from avian IAVs. These avian-like swIAVs emerged in Europe in 1979 and spread to Asia in 1993 (*10*–*13*). Apart from antigenic evolution in the HA, IAVs also can evolve via exchange of gene segments with other IAVs of different subtypes or clades infecting the same cell, called reassortment (*14*), which frequently occurs in pigs. A reassortant IAV with an antigenically novel HA and the capacity to infect and spread in humans could cause a pandemic.

Since 2010, 35 zoonotic infections with H1 swIAVs were reported in North America and 10 in Europe (*15**–**17*; Parys et al., unpub. data). Human population immunity is a major factor determining the pandemic risk for swIAVs. Hemagglutination inhibiting (HI) and virus neutralizing (VN) antibodies in serum are accepted correlates of protection (*18*). Evaluating humans of different age groups for HI and VN antibody titers against a range of antigenically different swIAVs might help clarify the public health risk.

In a previous seroprevalence study for H3 swIAVs in humans from Luxembourg, we demonstrated a correlation with the nature of the swIAV and its relation to human IAVs on the one hand and the persons’ birth year on the other (*19*). A large comparative seroprevalence study for H1 swIAVs is lacking. Previous studies examined limited numbers of H1 swIAVs or samples or did not evaluate the relation between birth year and antibody responses (*12*,*13*,*20*–*25*). In addition, most studies were conducted before or during the 2009 pandemic, but the circulation of pH1N1 viruses in humans likely changed the serologic profile against H1 swIAVs. We assessed prevalence and titers of protective antibodies against all major H1 swIAV clades in various age groups in Belgium in 2017. We also examined the relation between antibodies against human-like swIAVs and their presumed human seasonal ancestor IAV. The results will help assess the public health risk for different H1 swIAVs.

## Materials and Methods

### Sample Collection

During August 2017–January 2018, a total of 549 anonymized serum samples were collected from immunocompetent persons with unknown influenza vaccination or infection history born during 1920–2017 at Ghent University Hospital (Ghent, Belgium). Samples included »6 per birth year with »1:1 ratio between male and female patients. Exclusion criteria included active oncologic disease or hematologic malignancies, immunosuppressive treatment, organ transplantation, admission to intensive care, and end-stage renal disease on dialysis treatment. This study was approved by the Commission for Medical Ethics of the Ghent University Hospital (approval no. 2017/0834).

### Viruses

Samples were evaluated for antibodies against 11 viruses representing 7 major H1 swIAV clades circulating in Europe, North America, and Asia; 2 human seasonal progenitor IAVs for European and North American human-like swIAVs; and 1 human seasonal IAV that circulated right before the pH1N1 virus (Table 1). We used epidemiologic data (*10*–*12*) and the H1 classification system (*9*) to select major H1 swIAV clades. We selected test viruses on the basis of amino acid homology and antigenic relatedness to currently circulating swIAVs of each clade. We selected the human progenitor IAVs based on the literature (*26*,*27*).

**Table 1:** Swine and human H1 influenza A virus strains used in hemagglutination inhibition and virus neutralization assays of human serum samples, Belgium*

Virus strain	Abbreviation	Subtype	Colloquial name H1	H1 clade	H1 GenBank Accession no.
A/swine/Gent/28/2010	swG10	H1N1	European avian-like	1C.2.1	KP406525
A/swine/Hong Kong/2032/2011	swHK11	H1N1	Asian avian-like	1C.2.3	KM028543
A/Taiwan/1/86	TW86	H1N1	Human seasonal	1B.1-like	X17224
A/swine/Gent/26/2012	swG12	H1N2	European human-like	1B.1.2.1	KP406526
A/New Caledonia/20/99	NC99	H1N1	Human seasonal	1B.2-like	DQ508857
A/swine/Alabama/A01104091/2016	swAL16	H1N2	North American human-like δ1a	1B.2.2.1	KX247675
A/swine/Illinois/A01047020/2010	swIL10	H1N2	North American human-like δ1b	1B.2.2.2	JQ756323
A/swine/Oklahoma/A01290605/2013	swOK13	H1N1	North American human-like δ1b	1B.2.2.2	KF791395
A/Brisbane/59/2007	BR07	H1N1	Human seasonal	1B.2-like	CY058487
A/swine/Ohio/511445/2007	swOH07	H1N1	North American classical swine γ	1A.3.3.3	EU604689
A/California/04/2009	CA09	pH1N1	2009 pandemic	1A.3.3.2	FJ966082

*Viruses are ordered chronologically according to the year of circulation of the selected human test viruses TW86, NC99, BR07, and CA09; horizontal rules within table represent grouping of epidemiologically related human and swine influenza A viruses, with the oldest (ancestor) virus mentioned first.

We downloaded nucleotide sequences of the viruses’ HA1, the main target of neutralizing antibodies, from Genbank and translated these to amino acids. We used the MUSCLE algorithm for sequence alignment and the Jones-Taylor-Thornton model and nearest-neighbor-interchange heuristic method to construct maximum-likelihood trees in MEGA7 (*28*). We determined the percent of amino acid homology between test viruses and numbers of identical amino acids in presumed antigenic sites (*29*) with MEGA7 and R version 3.2.2 (*30*).

We obtained North American swIAVs and corresponding swine serum from the U.S. Department of Agriculture-Agricultural Research Service. We obtained human seasonal IAVs and corresponding ferret serum from Francis Crick Institute (London, UK), and Asian swIAV from Hong Kong University (Hong Kong). We antigenically characterized test viruses in cross-HI and cross-VN assays with postvaccination swine serum for swIAVs or postinfection ferret serum for human seasonal IAVs. Because serum against A/Brisbane/59/2007 was not available, we used ferret serum against A/Egypt/10/2007 instead; the HA sequence is identical in both. We propagated viruses in MDCK cells; all passages were <6. We calculated antigenic distances from HI and VN titers as described previously (*31*) and converted these into antigenic dendrograms by using the neighbor-joining method in MEGA7. One antigenic unit represents a 2-fold difference in HI or VN titer.

### Serologic Assays

We tested individual samples in HI assays and pooled samples per birth year in VN assays for antibodies against each test virus. Both assays were performed according to standard procedures (*32*,*33*). We expressed antibody titers for HI as the reciprocal of the highest serum dilution showing complete hemagglutination inhibition of 4 hemagglutinating units of virus or, for VN, 50% neutralization of 100 TCID_50_ (50% tissue culture infective doses) of virus. The starting dilution was 1:20, and we considered a titer of ≥40 positive.

### Statistical Analyses

We calculated geometric mean titers (GMTs) and 95% CIs for HI and VN antibody titers of samples from each birth decade against each test virus by using log_2_-transformed data. Samples with a titer <20 were assigned a titer of 10. For non-stratified data, we calculated Spearman correlation coefficients (CCs) between HI titers or between VN titers against different viruses. We used Kruskal-Wallis and Mann-Whitney U tests to compare antibody titers between age groups for a certain virus or between viruses for a certain age group. We used Fisher exact test to compare proportions of positive samples. For all statistical tests, we applied Bonferroni adjustment of the p values and we considered corrected p values of <0.05 statistically significant. We performed all analyses by using R version 3.2.2.

## Results

### Genetic and Antigenic Relatedness Between Test Viruses

**Figure 1:**
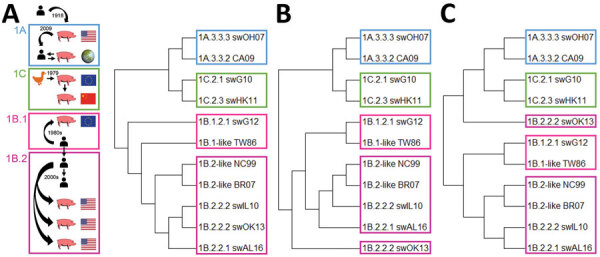
Epidemiologic, phylogenetic, and antigenic relationship between influenza A test viruses from classical swine lineage 1A, human seasonal lineage 1B, and Eurasian avian lineage 1C. A) Schematic representation of the H1 IAV epidemiology and maximum-likelihood neighbor-joining phylogenetic tree of the HA1 of representative test viruses. B) Antigenic dendrogram based on antigenic distances in cross-hemagglutination inhibition assays. C) Antigenic dendrogram based on antigenic distances in cross-virus neutralization assays. IAV, influenza A virus.

We tested samples for antibodies against 11 IAVs from the classical swine 1A, human seasonal 1B, or Eurasian avian 1C lineage. HA1 aa sequence homology between viruses of different lineages was <75% with 19–35/50 identical amino acids in presumed antigenic sites. Classical swine and avian-like IAVs were phylogenetically most closely related (Figure 1, panel A; Table 2). Within-lineage HA1 aa homology was 82%–97%, with 36–49 identical amino acids in antigenic sites. Human-like swIAVs and their presumed human seasonal progenitor IAV shared 90%–94% aa in the HA1 and 36–42 aa in antigenic sites.

**Table 2:** Percentage amino acid homology (lower left) and number of identical amino acids out of 50 aa in presumed antigenic sites (upper right) (*28*) between hemagglutinin 1 of human and swine H1 influenza A viruses used as test viruses in hemagglutination inhibition and virus neutralization assays*

Virus strain	Eurasian avian			Human seasonal								Classical swine	
	swG10	swHK11		TW86†	swG12	NC99†	swAL16	swIL10	swOK13	BR07†		swOH07	CA09
	Europe 1C.2.1	Asia 1C.2.3		1B.1-like	Europe 1B.1.2.1	1B.2-like	N. Am. 1B.2.2.1	N. Am. 1B.2.2.2	N. Am. 1B.2.2.2	1B.2-like		N. Am. 1A.3.3.3	World 1A.3.3.2
swG10	–	49		22	19	23	24	23	23	23		32	34
swHK11	96.0	–		23	20	23	25	23	23	23		33	35
TW86	73.4	72.2		–	41	41	38	36	32	38		23	23
swG12	69.1	68.5		89.6	–	35	33	33	32	32		21	22
NC99	73.3	72.1		93.9	85.9	–	42	42	36	47		24	24
swAL16	71.5	71.2		89.3	81.6	91.4	–	45	40	40		23	27
swIL10	72.1	71.5		89.6	83.4	94.2	92.9	–	43	42		23	25
swOK13	72.0	72.0		86.8	81.8	90.2	89.2	94.8	–	36		20	24
BR07	72.7	71.5		90.8	83.7	96.9	89.9	92.3	88.6	–		24	24
swOH07	74.0	74.3		74.3	71.6	73.0	72.4	71.8	70.5	73.6		–	44
CA09	73.4	72.5		73.4	70.9	72.4	73.9	72.1	71.1	72.4		90.8	–

*Viruses are ordered chronologically according to the year of circulation of the selected human test viruses TW86, NC99, BR07, and CA09; horizontal rules within table represent grouping of epidemiologically related human and swine influenza A viruses, with the oldest (ancestor) virus mentioned first. Complete isolate names are provided in Table 1. IAV, influenza A virus; N. Am., North America; –, not applicable. †Human IAV that no longer circulates; TW86 is the presumed human precursor for human-like H1 swine IAVs in Europe; NC99 is the presumed human precursor for human-like H1 swine IAVs in North America; BR07 is a human seasonal IAV that circulated right before the influenza A(H1N1)pdm09 virus; the influenza A(H1N1)pdm09 virus, represented by CA09, is circulating in both humans and swine worldwide.

Antigenic dendrograms based on cross-HI and cross-VN assays showed similar trends to the phylogenetic tree, except for swOK13 (Tables 3–5; Figure 1). This North American human-like δ1b swIAV (1B.2.2.2) clustered separately from other IAVs of its lineage, including its presumed human ancestor, NC99.

**Table 3:** Cross-reactivity between human and swine H1 influenza A viruses in hemagglutination inhibition assay*

Virus strain	Eurasian avian			Human seasonal								Classical swine	
	swG10	swHK11		TW86†	swG12	NC99†	swAL16	swIL10	swOK13	BR07†		swOH07	CA09
	Europe 1C.2.1	Asia 1C.2.3		1B.1-like	Europe 1B.1.2.1	1B.2-like	N. Am. 1B.2.2.1	N. Am. 1B.2.2.2	N. Am. 1B.2.2.2	1B.2-like		N. Am. 1A.3.3.3	World 1A.3.3.2
swG10	**640**	40		<20	<20	<20	<20	<20	<20	<20		<20	20
swHK11	640	**640**		<20	<20	<20	<20	<20	<20	<20		40	40
TW86	<20	<20		**1,280**	160	20	<20	<20	<20	<20		<20	<20
swG12	20	<20		160	**1,280**	20	20	<20	<20	<20		<20	<20
NC99	<20	<20		<20	<20	**640**	<20	<20	<20	80		<20	<20
swAL16	<20	<20		<20	<20	160	**320**	20	<20	20		<20	<20
swIL10	<20	<20		<20	<20	40	40	**320**	<20	160		<20	<20
swOK13	<20	<20		<20	<20	<20	<20	<20	**640**	<20		<20	<20
BR07	<20	<20		<20	<20	40	<20	<20	<20	**1,280**		<20	<20
swOH07	20	40		<20	<20	<20	<20	<20	20	<20		**1,280**	640
CA09	20	20		<20	<20	<20	<20	<20	<20	<20		160	**1,280**
*Viruses are ordered chronologically according to the year of circulation of the selected human test viruses TW86, NC99, BR07, and CA09; horizontal rules within table represent grouping of epidemiologically related human and swine influenza A viruses, with the oldest (ancestor) virus mentioned first. Complete isolate names are provided in Table 1. Bold indicates HI titer against the homologous virus. IAV, influenza A virus; HI, hemagglutination inhibition; N. Am., North America. †Human IAV that no longer circulates; TW86 is the presumed human precursor for human-like H1 swine IAVs in Europe; NC99 is the presumed human precursor for human-like H1 swine IAVs in North America; BR07 is a human seasonal IAV that circulated right before the influenza A(H1N1)pdm09 virus; the influenza A(H1N1)pdm09 virus, represented by CA09, is circulating in both humans and swine worldwide.													

**Table 5:** Antigenic distance in units between human and swine H1 IAVs in cross-hemagglutination inhibition assays (upper right) and cross-virus neutralization assays (lower left)*

Virus strain	Eurasian avian			Human seasonal								Classical swine	
	swG10	swHK11		TW86†	swG12	NC99†	swAL16	swIL10	swOK13	BR07†		swOH07	CA09
	Europe 1C.2.1	Asia 1C.2.3		1B.1-like	Europe 1B.1.2.1	1B.2-like	N. Am. 1B.2.2.1	N. Am. 1B.2.2.2	N. Am. 1B.2.2.2	1B.2-like		N. Am. 1A.3.3.3	World 1A.3.3.2
swG10	–	1.67		4.96	4.67	4.49	4.15	4.30	4.24	4.69		4.41	4.23
swHK11	1.93	–		5.20	5.03	4.77	4.53	4.69	4.50	4.92		3.75	3.82
TW86	4.84	4.98		–	2.12	4.77	4.54	4.79	4.80	5.14		5.63	5.54
swG12	4.87	5.09		2.31	–	4.77	4.33	4.74	4.77	5.14		5.65	5.53
NC99	4.65	4.37		4.72	4.85	–	2.62	3.04	4.35	2.74		5.19	5.08
swAL16	4.62	4.53		4.79	4.71	1.75	–	2.65	3.99	3.67		4.91	4.75
swIL10	4.95	4.93		5.27	5.19	2.50	2.29	–	4.11	2.75		5.09	4.92
swOK13	4.34	4.44		4.79	4.83	4.47	4.44	4.63	–	4.56		4.65	4.81
BR07	4.77	4.36		4.97	5.06	1.91	2.75	2.75	4.63	–		5.35	5.26
swOH07	4.65	3.48		5.07	5.21	4.71	4.80	5.12	4.73	4.44		–	1.43
CA09	4.59	3.91		4.89	5.28	5.09	5.01	5.33	4.53	5.17		2.06	–
*Viruses are ordered chronologically according to the year of circulation of the selected human test viruses TW86, NC99, BR07, and CA09; horizontal rules within table represent grouping of epidemiologically related human and swine influenza A viruses, with the oldest (ancestor) virus mentioned first. Complete isolate names are provided in Table 1. IAV, influenza A virus; N. Am., North America; – not applicable. †Human IAV that no longer circulates; TW86 is the presumed human precursor for human-like H1 swine IAVs in Europe; NC99 is the presumed human precursor for human-like H1 swine IAVs in North America; BR07 is a human seasonal IAV that circulated right before the influenza A(H1N1)pdm09 virus; the influenza A(H1N1)pdm09 virus, represented by CA09, is circulating in both humans and swine worldwide.													

### Seroreactivity against Human Seasonal IAVs

**Figure 2:**
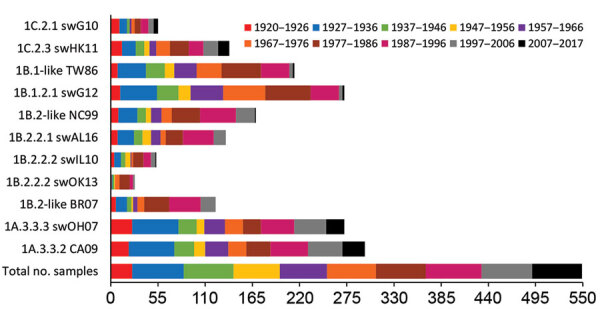
Number of positive human serum samples in the hemagglutination inhibition assay (titer >40) for each test virus compared with the total number of samples tested per birth cohort. Birth cohorts are represented as different colors. During August 2017–January 2018, a total of 549 serum samples were collected from immunocompetent persons in Belgium.

**Figure 3:**
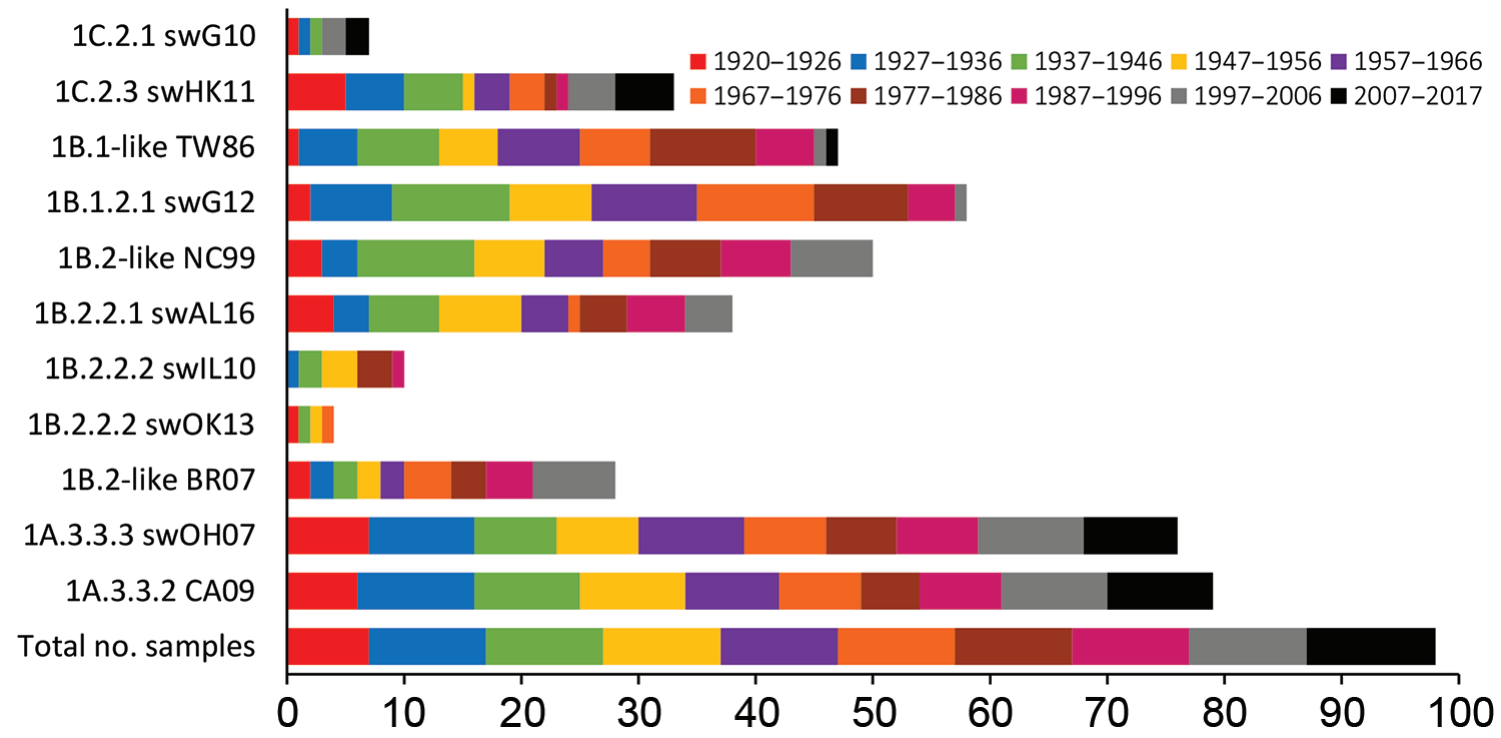
Number of positive human serum samples in the virus neutralization assay (titer >40) for each test virus compared with the total number of samples per birth cohort. Birth cohorts are represented as different colors. During August 2017–January 2018, a total of 549 serum samples were collected from immunocompetent persons in Belgium and pooled per year of birth (n = 98).

We tested human serum samples against human seasonal IAVs related to 1B swIAVs from 1986 (TW86), 1999 (NC99), and 2007 (BR07) to evaluate a person’s potential exposure to or vaccination with these IAVs. Overall, 39% were seropositive for TW86, 31% for NC99, and 22% for BR07 in HI and 48% were seropositive for TW86, 51% for NC99, and 29% for BR07 in VN (Figures 2, 3). Seroprevalences and GMTs against TW86 were highest for persons born during 1977–1986 and lowest for the 2 youngest groups, those born during 1997–2017 (Tables 6, 7). For NC99 and BR07, HI responses were highest for those born during 1987–1996, and VN responses were highest for those born during 1937–1946 (NC99 only) and 1997–2006. Persons born during 2007–2017 had minimal responses. Antibody responses against human seasonal IAVs were related to birth year and the year of virus isolation, with peak responses in persons born right before the virus circulated and lowest responses in persons born afterwards.

**Table 6:** Geometric mean of hemagglutination inhibition antibody reactivity against different H1 influenza A viruses of humans and swine in different age groups of the population, 2017–2018, Belgium*

Birth year range (age, y‡)	No.	Eurasian avian			Human seasonal									Classical swine	
		swG10	swHK11		TW86†	swG12	NC99†	swAL16	swIL10	swOK13		BR07†		swOH07	CA09
		Eur. 1C.2.1	Asia 1C.2.3		1B.1-like	Eur. 1B.1.2.1	1B.2-like	N. Am. 1B.2.2.1	N. Am. 1B.2.2.2	N. Am. 1B.2.2.2	1B.2-like			N. Am. 1A.3.3.3	World 1A.3.3.2
1920–26 (91–97)	25	24 (16–38)	38 (24–60)		21 (15–31)	26 (18–39)	25 (18–35)	24 (17–35)	14 (11–17)	12 (10–16)	19 (14–28)			236 (147–379)	125 (70–222)
1927–36 (81–90)	60	15 (12–19	20 (16–26)		38 (29–51)	49 (39–61)	24 (20–28)	20 (17–24)	15 (13–18)	12 (11–13)	18 (15–20)			116 (87–155)	98 (73–132)
1937–46 (71–80)	58	12 (10–13)	15 (12–18)		26 (19–36)	30 (22–40)	16 (13–20)	14 (12–17)	12 (11–14)	12 (10–13)	12 (11–13)			23 (17–31)	28 (20–38)
1947–56 (61–70)	54	10 (10–11)	12 (11–14)		17 (13–23)	19 (15–25)	14 (12–17)	16 (13–20)	12 (11–14)	10 (10–11)	11 (10–13)			15 (12–19)	18 (14–23)
1957–66 (51–60)	55	11 (10–13)	14 (12–17)		31 (22–42)	55 (42–72)	17 (13–21)	16 (14–20)	11 (10–12)	11 (10–12)	13 (11–16)			25 (20–32)	32 (24–44)
1967–76 (41–50)	57	12 (11–14)	18 (14–23)		34 (26–44)	76 (60–97)	16 (13–20)	14 (12–16)	12 (10–13)	12 (11–14)	14 (12–17)			23 (18–29)	25 (19–33)
1977–86 (31–40)	58	14 (12–17)	25 (19–32)		97 (68–138)	87 (69–110)	42 (31–57)	22 (17–28)	17 (14–21)	18 (15–22)	29 (23–38)			27 (22–34)	39 (28–54)
1987–96 (21–30)	65	13 (11–16)	19 (16–23)		45 (32–65)	37 (28–50)	42 (32–54)	35 (28–44)	15 (12–17)	13 (12–15)	40 (31–52)			37 (28–48)	54 (39–74)
1997–2006 (11–20)	59	14 (12–17)	21 (17–27)		13 (11–15)	13 (11–15)	22 (18–28)	19 (15–23)	14 (12–16)	11 (10–12)	18 (15–23)			48 (35–66)	73 (49–109)
2007–17 (0–10)	58	13 (11–16)	18 (13–24)		11 (10–12)	11 (10–12)	10 (10–11)	10 (10–10)	11 (10–12)	10 (10–10)	10 (10–10)			24 (18–33)	35 (24–53)
All (0–97)	549	13 (13–14)	18 (17–20)		28 (25–31)	33 (30–36)	21 (19–23)	18 (17–19)	13 (13–14)	12 (12–12)	17 (16–18)			35 (31–38)	42 (37–47)
*Values expressed represent geometric mean hemagglutination inhibition titers (95% CI). Eur., Europe; N. Am., North America. †Human IAV that no longer circulates; TW86 is the presumed human precursor for human-like H1 swine IAVs in Europe; NC99 is the presumed human precursor for human-like H1 swine IAVs in North America; BR07 is a human seasonal IAV that circulated right before the influenza A(H1N1)pdm09 virus; the influenza A(H1N1)pdm09 virus, represented by CA09, is circulating in both humans and swine worldwide. ‡Age at the end of 2017.															

**Table 7:** Geometric mean of virus neutralization titers and antibody reactivity against different human and swine H1 influenza A viruses in different age groups of the population, 2017–2018, Belgium*

Birth year range (age, y‡)	Eurasian avian				Human seasonal								Classical swine	
	swG10		swHK11		TW86†	swG12	NC99†	swAL16	swIL10	swOK13	BR07†		swOH07	CA09
	Europe 1C.2.1		Asia 1C.2.3		1B.1-like	Europe 1B.1.2.1	1B.2-like	N. Am. 1B.2.2.1	N. Am. 1B.2.2.2	N. Am. 1B.2.2.2	1B.2-like		N. Am. 1A.3.3.3	World 1A.3.3.2
1920–6 (91–97)	24 (8–68)		67 (24–187)		14 (8–25)	17 (9–33)	29 (13–65)	29 (13–62)	16 (9–28)	18 (11–32)	19 (10–37)		170 (54–533)	111 (49–254)
1927–36 (81–90)	13 (9–19)		34 (19–58)		38 (26–57)	57 (39–84)	27 (21–34)	26 (17–40)	14 (10–20)	19 (12–27)	20 (12–33)		93 (63–135)	101 (79–128)
1937–46 (71–80)	14 (8–23)		31 (18–54)		68 (39–118)	98 (64–149)	53 (41–68)	36 (23–55)	22 (13–35)	17 (11–26)	34 (23–50)		42 (25–69)	85 (55–133)
1947–56 (61–70)	11 (9–13)		19 (12–30)		29 (16–54)	59 (38–93)	36 (22–60)	59 (34–102)	30 (21–44)	18 (12–26)	19 (12–30)		47 (27–83)	73 (45–119)
1957–66 (51–60)	10 (10–10)		20 (13–32)		52 (37–72)	80 (51–124)	40 (28–58)	36 (25–53)	11 (9–13)	11 (9–13)	26 (14–47)		52 (40–67)	72 (45–116)
1967–76 (41–50)	11 (9–13)		23 (14–36)		41 (22–76)	109 (76–158)	27 (16–47)	16 (10–26)	11 (9–13)	13 (9–19)	24 (13–45)		40 (22–74)	38 (22–66)
1977–86 (31–40)	10 (10–10)		14 (9–21)		86 (55–137)	81 (47–138)	38 (22–65)	27 (15–48)	18 (11–30)	13 (10–17)	23 (14–37)		35 (19–65)	31 (16–60)
1987–96 (21–30)	10 (10–10)		15 (10–24)		41 (20–83)	34 (18–64)	46 (25–84)	43 (23–78)	13 (9–21)	11 (9–13)	35 (21–58)		46 (31–67)	47 (26–86)
1997–2006 (11–20)		15 (9–26)	31 (15–64		17 (10–27)	15 (10–22)	53 (29–99)	33 (20–55)	10 (10–10)	11 (9–14)	40 (23–70)		117 (54–256)	149 (58–379)
2007–17 (0–10)	18 (9–37)		37 (15–90)		11 (9–15)	12 (9–15)	11 (9–14)	10 (10–10)	10 (10–10)	10 (10–10)	10 (10–10)		70 (26–189)	88 (35–221)
All (0–97)	13 (11–14)		25 (21–31)		33 (28–40)	44 (36–54)	33 (28–39)	28 (24–33)	14 (13–16)	13 (12–15)	23 (20–27)		60 (50–73)	71 58–86)

*Values expressed represent geometric mean virus neutralization titers of pooled serum samples per birth year (95% CI). Boldface indicates geometric mean VN titers of pooled serum samples per birth year. N. Am., North America. †Human IAV that no longer circulates; TW86 is the presumed human precursor for human-like H1 swine IAVs in Europe; NC99 is the presumed human precursor for human-like H1 swine IAVs in North America; BR07 is a human seasonal IAV that circulated right before the influenza A(H1N1)pdm09 virus; the influenza A(H1N1)pdm09 virus, represented by CA09, is circulating in both humans and swine worldwide. ‡Age at the end of 2017.

### Seroreactivity against swIAVs of the Eurasian Avian Lineage 1C

The major avian-origin swIAV clades are European avian-like 1C.2.1, represented by swG10, and Asian avian-like 1C.2.3, represented by swHK11. For swG10, 10% of all samples tested positive in HI and 7% in VN (Figures 2, 3). Seroprevalence was <20% and GMTs were <20 for all age groups except the oldest, those born during 1920–1926, with 40% seropositive in HI and GMTs for HI and VN of 24 (Tables 6, 7).

For swHK11, overall seroprevalence was 25% in HI and 34% in VN. As for swG10, responses against swHK11 were highest for those born during 1920–1926; 52% in HI, 72% in VN, and GMTs ≥38. Responses were minimal in both HI and VN for persons born during 1947–1956; 11% in HI, 10% in VN, and GMTs <20.

### Seroreactivity against swIAVs of the Human Seasonal Lineage 1B

European human-like swIAV swG12 (1B.1.2.1) represents the human-like H1 swIAV clade circulating in Belgium, and TW86 was selected as its presumed human ancestor virus. At least half of all samples tested positive for swG12, 50% in HI and 59% in VN (Figures 2, 3). We noted statistically significant differences in seroprevalences and GMTs, which were higher (62% in HI and 74% in VN; GMTs ≥44) in persons born before 1996 than in persons born during 1997–2017 (5% in HI and in VN; GMTs <20; p<0.001) (Tables 6, 7). GMTs peaked (≥87) in HI in those born during 1977–1986 and in VN for those born during 1967–1976. Results for swG12 were similar to those for its presumed human ancestor virus, TW86.

North American human-like δ H1 swIAVs (1B.2) result from the introduction of a human IAV in the early 2000s, and we selected NC99 as their presumed human ancestor. For the most prevalent δ1 clade (1B.2.2), swAL16 represents subclade δ1a (1B.2.2.1), whereas swIL10 and swOK13 represent subclade δ1b (1B.2.2.2).

Among samples, 24% tested positive for swAL16 in HI and 39% in VN (Figures 2, 3). Seroprevalences and GMTs were highest in those born during 1987–1996 in HI (55%; GMT 35) and in those born during 1947–1956 in VN (70%; GMT 59), but no antibodies against swAL16 were detected in the youngest group, those born during 2007–2017 (Tables 6, 7). Like for European human-like virus swG12 (1B.1.2.1), antibody responses against North American δ1a virus swAL16 (1B.2.2.1) resembled those against its presumed human ancestor virus, NC99.

For the δ1b swIAVs (1B.2.2.2), <10% were seropositive (swIL10, 10% in HI and VN; swOK13, 5% in HI and 4% in VN) (Figures 2, 3). We did not see statistically significant differences in seroprevalences between the 2 δ1b swIAVs or between age groups, with following exceptions. HI-seroprevalence of those born during 1927–1936 was statistically significantly higher for swIL10 (13%) than for swOK13 (2%; p<0.04). HI seroprevalence for swOK13 of those born during 1977–1986 was statistically significantly higher (21%) compared with groups born during 1927–1936 (2%), 1957–1966 (0), and 2007–2017 (0; p<0.04). GMTs were <20 in all age groups except those born during 1937–1956, who had VN GMTs of 22–30 against swIL10 (Tables 6, 7). Unlike the other 2 human-like swIAVs tested, responses against δ1b swIAVs (1B.2.2.2) did not concur with those against the presumed human ancestor virus NC99. Responses against swIL10 and swOK13 were generally statistically significantly lower than against NC99 (p<0.05).

### Seroreactivity against swIAVs of the Classical Swine Lineage 1A

We used swOH07 as reference virus to evaluate antibody responses against classical swine virus clade γ (1A.3.3.3) and CA09 as reference virus to evaluate classical swine virus clade pH1N1 (1A.3.3.2), which derived its HA from γ swIAVs. Overall, ≥50% of the samples tested positive for swOH07 (50% in HI; 78% in VN) and CA09 (54% in HI; 81% in VN), with high seroprevalences in all age groups (36%–100% in HI; 50%–100% in VN), except in those born during 1947–1956 in HI (swOH07, 17%; CA09, 24%) (Figures 2, 3). HI titers peaked in the 2 oldest groups, those born during 1920–1936; VN titers peaked in the 2 oldest groups and in those born during 1997–2006 (Tables 6, 7). No statistically significant difference was noted in responses against classical swine γ (1A.3.3.3) and pH1N1 (1A.3.3.2) IAVs.

### Correlations Between Antibody Titers against Human Seasonal and Swine IAVs

Antibody titers against epidemiologically related human and swine IAVs were highly correlated for classical swine viruses swOH07 (1A.3.3.3) and CA09 (1A.3.3.2), European human-like swIAV swG12 (1B.1.2.1) and human ancestor IAV TW86, North American human-like δ1a swIAV swAL16 (1B.2.2.1) and human ancestor IAV NC99, and European and Asian avian-like swIAVs swG10 (1C.2.1) and swHK11 (1C.2.3) (CC = 0.68–0.86 in HI; CC = 0.63–0.77 in VN; Table 8). Of note, titers against avian-like and classical swine IAVs also were strongly correlated (CC = 0.55–0.68 in HI; CC = 0.49–0.67 in VN). In contrast, CCs were low between titers against North American human-like δ1b viruses swIL10 and swOK13 (1B.2.2.2) and human ancestor virus NC99 (0.42–0.43 in HI; 0.30–0.39 in VN [the first value of which is not statistically significant]).

**Table 8:** Spearman correlation coefficients between hemagglutination inhibition antibody titers against human and swine H1 influenza A viruses (upper right) and between virus neutralization antibody titers against human and swine H1 influenza A viruses (lower left)*

Virus strain	Eurasian avian			Human seasonal								Classical swine	
	swG10	swHK11		TW86†	swG12	NC99†	swAL16	swIL10	swOK13	BR07†		swOH07	CA09
	Europe 1C.2.1	Asia 1C.2.3		1B.1-like	Europe 1B.1.2.1	1B.2-like	N. Am. 1B.2.2.1	N. Am. 1B.2.2.2	N. Am. 1B.2.2.2	1B.2-like		N. Am. 1A.3.3.3	World 1A.3.3.2
swG10	–	0.68		NS	0.16	0.19	0.19	NS	0.26	0.18		0.56	0.55
swHK11	0.63	–		0.24	0.26	0.32	0.31	0.23	0.43	0.30		0.68	0.63
TW86	NS	NS		–	0.84	0.52	0.43	0.21	0.35	0.35		0.25	0.26
swG12	NS	NS		0.77	–	0.42	0.41	0.23	0.37	0.28		0.30	0.27
NC99	NS	NS		0.46	0.37	–	0.69	0.42	0.43	0.75		0.36	0.36
swAL16	NS	NS		NS	NS	0.71	–	0.53	0.38	0.58		0.43	0.39
swIL10	NS	NS		NS	0.37	0.39	0.52	–	0.37	0.45		0.30	0.26
swOK13	NS	NS		NS	NS	NS	NS	0.35	–	0.39		0.33	0.34
BR07	NS	NS		NS	NS	0.76	0.57	NS	NS	–		0.30	0.31
swOH07	0.55	0.67		NS	NS	NS	NS	NS	NS	NS		–	0.86
CA09	0.49	0.62		NS	NS	NS	NS	NS	NS	NS		0.74	–
*Viruses are ordered chronologically according to the year of circulation of the selected human test viruses TW86, NC99, BR07, and CA09; horizontal rules within table represent grouping of epidemiologically related human and swine influenza A viruses, with the oldest (ancestor) virus mentioned first. Complete isolate names are provided in Table 1. N. Am., North America; NS, not significant; –, not applicable. †Human IAV that no longer circulates; TW86 is the presumed human precursor for human-like H1 swine IAVs in Europe; NC99 is the presumed human precursor for human-like H1 swine IAVs in North America; BR07 is a human seasonal IAV that circulated right before the influenza A(H1N1)pdm09 virus; the influenza A(H1N1)pdm09 virus, represented by CA09, is circulating in both humans and swine worldwide.													

## Discussion

Our results show that serum antibody responses of immunocompetent persons in Belgium against major H1 swIAV clades depend on the swIAV tested and its relation to human seasonal IAVs and the person’s birth year. Overall seroprevalences were high (≥50%) for classical swine (1A.3.3.2, 1A.3.3.3) and for European human-like (1B.1.2.1) swIAVs, intermediate (≥24%) for North American human-like δ1a (1B.2.2.1) and Asian avian-like (1C.2.3) swIAVs, and low (<10%) for North American human-like δ1b (1B.2.2.2) and European avian-like (1C.2.1) swIAVs. Our results are consistent with previous studies that aimed to compare antibody responses in nonswine workers with those in persons with frequent swine contact (*7*,*20*–*25*), although those studies examined only a limited number of swIAV clades or samples. Overall, most previous studies showed lower seroprevalences for Asian avian-like (2%–10%) and European avian-like (0–5%) swIAVs in the general population or in nonswine workers (*13*,*20*,*22*–*24*). A 2010 study in the United Kingdom also found a lower seroprevalence of 11% for a European human-like (1B.1.2.1) swIAV (*24*). The major difference between our study and studies conducted before or during the 2009 pandemic is the lower seroprevalence of 3%–15% for classical swine IAVs in previous studies (*13*,*20*,*22*–*24*). The circulation of pH1N1 viruses (1A.3.3.2) likely contributes to increased seroprevalence rates against these related classical swine IAVs. In our study, the oldest group, those born during 1920–1926 who are 91–97 years of age, had the highest antibody responses against H1 swIAVs of classical swine (1A.3.3) and avian-like (1C.2) lineages, for which antibody titers were correlated (*13*,*20*,*21*). Responses against human seasonal IAVs and related European and North American δ1a human-like H1 swIAVs (1B) generally were highest in those born during 1977–1996, who are 21–40 years of age, and lowest in those born during 1996–2017, who are 0–20 years of age. Responses against North American δ1b human-like H1 swIAVs (1B.2.2.2) generally were low across all age cohorts.

Antibody responses against past human seasonal IAVs TW86, NC99, and BR07 generally peaked in persons born near the time during which the respective IAV or similar viruses circulated, whereas responses were low in most persons born after. Within an age group, responses generally were highest against an antigenic representative of the virus encountered first. These findings concur with the theory of antigenic seniority: humans are expected to have antibodies against human seasonal IAVs that circulated after their birth, with highest responses against the virus encountered first. Antigenic seniority likely occurs because of periodic boosting of these antibodies by subsequent exposures to related human seasonal IAVs (*4*,*34*,*35*). Antibody titers against European human-like swIAV swG12 (1B.1.2.1) and North American human-like δ1a swIAV swAL16 (1B.2.2.1) concur with those against their respective human ancestor viruses TW86 and NC99 because of close antigenic relationship to their ancestor IAV. Overall high seroprevalences against pH1N1 virus CA09 (1A.3.3.2) and antigenically closely related classical swine γ virus swOH07 (1A.3.3.3) can be explained by recent exposure to currently circulating pH1N1 viruses. Because the oldest persons were born during 1920–1936, when human IAVs closely related to the 1918 pandemic virus, the ancestor of classical swine IAVs, circulated, they could have had cross-reactive antibodies against classical swine IAVs before 2009. These antibodies might have been boosted by later exposure to pH1N1 viruses, which might account for the high responses in this group (*6*,*7*). Consistent with results for serum samples collected after pH1N1 virus infection in a previous study (*21*), cross-reactivity was higher against the Asian than against the European avian-like H1 swIAV, which differ by only 1 aa in antigenic sites (Table 2). Whether this single amino acid mutation is the reason for the difference in seroprevalence is still unknown (*36*,*37*).

European human-like (1B.1.2.1) and North American human-like δ1a (1B.2.2.1) H1 swIAVs are antigenically more closely related to their human ancestor than North American human-like δ1b (1B.2.2.2) H1 swIAVs (Figure 1; Tables 3, 4). North American human-like δ H1 swIAVs (1B.2) have been shown to drift 4 times faster than European human-like H1 swIAVs (1B.1). Increased antigenic diversity of the former since 2008 has led to the emergence of swIAVs that are antigenically distinct from the human precursors, mainly within the δ1b subclade (*3*,*27*). This evolution can explain the recognition of selected European human-like and North American human-like δ1a but not North American human-like δ1b H1 swIAVs by human serum samples. Because the human ancestor IAVs no longer circulate in humans, swine can be considered a reservoir for old human IAVs. Seroprevalences for European human-like and North American human-like δ1a H1 swIAVs are expected to decrease over time because the youngest age groups were never exposed to these human IAVs. On the basis of our results, we estimate that it could take <80 years for the population to become fully susceptible.

**Table 4:** Cross-reactivity between human and swine H1 IAVs in virus neutralization assay*

Virus strain	Eurasian avian			Human seasonal								Classical swine	
	sG10	swHK11		TW86†	swG12	NC99†	swAL16	swIL10	swOK13	BR07†		swOH07	CA09
	Europe 1C.2.1	Asia 1C.2.3		1B.1-like	Europe 1B.1.2.1	1B.2-like	N. Am. 1B.2.2.1	N. Am. 1B.2.2.2	N. Am. 1B.2.2.2	1B.2-like		N. Am. 1A.3.3.3	World 1A.3.3.2
swG10	**1,920**	40		<20	<20	<20	<20	<20	<20	<20		<20	20
swHK11	640	**640**		<20	<20	30	<20	<20	<20	40		60	80
TW86	<20	<20		**1,920**	320	40	<20	<20	<20	<20		<20	30
swG12	<20	<20		120	**3,840**	20	20	<20	<20	<20		<20	<20
NC99	<20	<20		<20	<20	**1,280**	30	80	<20	160		<20	<20
swAL16	<20	<20		<20	<20	640	**960**	80	<20	160		<20	<20
swIL10	<20	<20		<20	<20	120	160	**3,840**	<20	320		<20	<20
swOK13	<20	<20		<20	<20	<20	<20	20	**1,280**	<20		<20	20
BR07	<20	<20		<20	<20	240	<20	60	<20	**2,560**		<20	<20
swOH07	<20	60		<20	20	20	<20	<20	<20	120		**1,920**	960
CA09	<20	<20		<20	<20	<20	<20	<20	<20	<20		160	**3,840**
*Viruses are ordered chronologically according to the year of circulation of the selected human test viruses TW86, NC99, BR07, and CA09; horizontal rules within table represent grouping of epidemiologically related human and swine influenza A viruses, with the oldest (ancestor) virus mentioned first. Complete isolate names are provided in Table 1. Bold indicates VN titer against the homologous virus IAV, influenza A virus; VN, virus neutralization; N. Am., North America. †Human IAV that no longer circulates; TW86 is the presumed human precursor for human-like H1 swine IAVs in Europe; NC99 is the presumed human precursor for human-like H1 swine IAVs in North America; BR07 is a human seasonal IAV that circulated right before the influenza A(H1N1)pdm09 virus; the influenza A(H1N1)pdm09 virus, represented by CA09, is circulating in both humans and swine worldwide.													

Seroprevalences of immunocompetent persons in Belgium for swIAVs representing major H1 swIAV clades suggest that North American human-like δ1b (1B.2.2.2) and European avian-like (1C.2.1) H1 swIAVs currently pose the highest risk to public health. North American human-like δ1b (1B.2.2.2) swIAVs rapidly drifted away from its human ancestor, whereas European avian-like (1C.2.1) swIAVs never circulated in humans. Seroprevalences of <10% for these viruses are comparable to 2%–19% against the pH1N1 virus right before the pandemic (*7*). Our results suggest that the risk of reintroduction of these H1 swIAVs in the human population might be higher than for H3 swIAVs, given that ≥20% of persons 0–100 years of age from Luxembourg tested seropositive for representative European and North American cluster IV H3 swIAVs in 2010 (*19*). Seroprevalences against the other currently circulating human-like H1 swIAV clades were higher than against 1B.2.2.2 and 1C.2.1, but these viruses, along with H3 swIAVs, also keep evolving in swine. As they continue to drift away from their human ancestor and population immunity wanes with lack of exposure, these viruses might also pose a risk to public health soon.

We evaluated human population immunity against H1 swIAVs on the basis of serum HI and VN antibodies, which are directed against the highly variable head region of the HA. We did not measure antibodies or T-cell responses against the HA stalk, the neuraminidase, or internal viral proteins, such as the nucleoprotein. Although these immune mechanisms are much less potent than neutralizing anti-HA antibodies, their targets are more conserved between IAVs of humans and swine (*38*–*42*). Therefore, persons with minimal antibody titers in our study still might have some degree of immunity and protection against zoonotic infection with swIAVs. Furthermore, population immunity is only one aspect determining the pandemic potential of swIAVs (*18*). Another factor is their ability to spread in humans, which is difficult to investigate (*43*). Our results stress the need for continuous surveillance and characterization of circulating swIAVs and frequent monitoring of humans for antibodies against these swIAVs.
